# Post Mortem Examination of a Dirt-Eater

**Published:** 1885-08

**Authors:** Howard J. Williams

**Affiliations:** Macon, Ga.; Macon, Ga.


### POST MORTEM EXAMINATION OF A DIRT-EATER.

BY DR. HOWARD J. WILLIAMS AND DR. M'HATTON.

Reported for THE ATLANTA MEDICAL AND SURGICAL JOURNAL.

The following *post mortem* may be of interest, as it may perhaps indicate the pathological changes to be found resulting from dirt-eating. The examination was made last November at the "South Georgia Conference Orphans' Home," near Macon. Dr. Holt, the attending physician, and Dr. Kenan Hall were present. The clinical history was very meagre, as the patient had arrived at the Home a few days before and his death was rather sudden. The patient, a confirmed dirt-eater, white, male, aged ten years, small for his age, not much emaciated, but profoundly anæmic, the skin presenting the peculiar chamois skin color common to this class of patients, had been sick only a few hours when death occurred. The *post mortem* was held twelve hours after death and rigor *mortis* was well established. Opening the abdominal cavity the peritoneum presented a pale glistening appearance, with a normal amount of fluid present. *Free grit* was found in peritoned cavity. The large intestine was bound down posteriorly and in some places matted together by old adhesions. The adhesions extended from the virmiform appendix to the rectum. The pressure of the grit in the cavity of the peritoneum accounts for the adhesions, perforation and escape of the grit causing peritonitis at some perod in the past history of the case. The intestines were distended with gas and contained a normal amount of feces. On the inner coat of the intestines there were several points of ecchymoses.

The stomach was distended with gas. The pylorus was decidedly thickened. (Thickening of the pylorus is often the result of local irritation, as the passage of foreign bodies, as in the present case.)

The liver was very much enlarged (about the size of an adult organ), edges rounded, a doughy aspect, and of a yellowish gray color. On section there were several layers which were vitreous and semi-transparent.

The spleen was enlarged, twice the size of the normal (adult) gland, thickened, homogeneous in appearance, on section, soft and doughy, the capsule thickened and adherent.

The kidneys were enlarged (the right larger than the left), most of the thickening being in the cortical substance, pale gray in color, the pyramids were distinct though pale, capsule non-adherent.

These organs had evidently undergone amyloid degeneration; the kidneys advanced only in the first stage.

Opening the thoracic cavity, the lungs were found non-adherent, a normal amount of fluid in the pleural cavity. The lower part of the upper lobe of the left lung on section presented a begining croupous pneumonia. The extreme free border of the lobe had gone into the third stage or stage of purulent infiltration. The remainder of the left and the entire right lung were normal, except a few spots of hypostatic pneumonia posteriorly.

In the pericardium there was no evidence of active inflammation, although there was present about a gill of fluid. Both ventricles of the heart were dilated. The left was filled with a soft. friable clot, which had no distinct layers, and the blood was darker than normal. This character of blood clot is often present in anæmic conditions, as scorbutus, poisoning by phosphoretic hydrogen and that class of diseases.

In regard to the immediate cause of death, we may say that, owing to the reduced condition of his vital powers, he would readily have yielded to any intercurrent disease, and we are convinced that the small focus of pneumonic infiltration was the immediate cause of death. The small spot of solidification presented sufficient obstacle to the blood current to cause, in the weakened condition of the heart, death by asthenia.

This patient, as is the case with all dirt-eaters, was very anæmic, and all the organs presented amyloid degeneration, which is common to profound anæmia from any chronic disease of the blood-making organs. As these dirt-eaters are anæmic, and as amyloid degeneration is common in protracted anæmia from whatever cause, it is natural that we should incline to the conclusion that amyloid degeneration is always associated with the habit, and in dirt-eating we have, perhaps, another cause of amyloid changes. Yet nothing positive can be decided without further observations, for these visceral changes may not be present in other individuals of the class.

That it is a vice of nutrition we all recognize, but the primary cause of the vice is not yet determined. Whether the depraved appetite is the effect of the disordered nutrition and the changes in the blood-making organs, or the disorders of nutrition and the anæmia attending the habit are caused by the appetite, is still doubtful.

The habit is a common disease, occurring in the West Indies, Africa, South America, and especially in the temperate and tropic zones. Once acquired it is never abandoned; change in climate and hygienic surroundings have no controlling influence on the vitiated appetite. Reports of similar cases and careful *post mortem* examinations would give light on this subject, of which so little is known.

